# Approaches to deorphanize secretome: Classical, computational, and next generation strategies to reveal ligand-receptor networks

**DOI:** 10.70401/EXO.2026.0008

**Published:** 2026-05-11

**Authors:** Myeonghoon Han, Norbert Perrimon

**Affiliations:** 1 Department of Genetics, Blavatnik Institute, Harvard Medical School, Boston, MA 02115, USA.; 2 Howard Hughes Medical Institute, Harvard Medical School, Boston, MA 02115, USA.

**Keywords:** Ligand, receptor, deorphanization, CRISPR screening, AlphaFold

## Abstract

Secreted proteins mediate intercellular and inter-organ communication and are essential for coordinating physiological processes across tissues. Advances in proteomics and proximity labeling have greatly expanded the catalog of circulating secreted factors; however, for many of these molecules, their cognate receptors and mechanisms of action remain unknown. This lack of receptor annotation represents a major bottleneck in understanding systemic signaling networks and translating secretome discoveries into biological insights. In this review, we summarize and evaluate the strengths and limitations of current strategies for deorphanizing secreted proteins, including 1) biochemical approaches such as affinity purification–mass spectrometry and crosslinking-based receptor capture, 2) genetic screening strategies in both *in vivo* and *in vitro* systems, including RNA interference and Clustered Regularly-Interspaced Short Palindromic Repeats (CRISPR)-based perturbation and activation platforms, and 3) computational frameworks based on AI-driven protein structure modeling. Finally, we outline future directions aimed at accelerating ligand–receptor identification, including multiplexed screening platforms, approaches to improve sensitivity for low-affinity interactions, synthetic biology tools that convert transient binding events into stable readouts, and integration with single-cell and spatial transcriptomic technologies. Together, these advances provide a roadmap for transforming classical ligand deorphanization into a scalable, context-aware framework for decoding inter-organ communication.

## 1. Introduction

Secreted proteins constitute a major class of circulating signaling molecules that mediate intercellular and inter-organ communication across metazoans^[1,2]^. These factors circulate through the blood and hemolymph in vertebrates and invertebrates, respectively. These secreted molecules, produced from specific tissues, act on distal target cells to coordinate physiology at the organismal level^[2]^. The secretome encompasses a diverse array of molecular entities, including classical hormones, cytokines, growth factors, small peptides, and metabolites^[3]^. These factors are produced constitutively or in response to environmental, metabolic, or cellular cues, enabling organisms to dynamically adapt to changes in nutrient availability^[4,5]^, stress^[6,7]^, development^[8,9]^, and disease states^[10–13]^.

The biological significance of secreted factors is most evident in endocrine regulation. For example, elevated circulating glucose triggers insulin secretion from pancreatic beta cells, which in turn orchestrates systemic glucose uptake and metabolic homeostasis, counterbalanced by glucagon secretion^[14]^. Beyond classical hormones, recent advances in proteomics and labeling technologies have dramatically expanded the catalog of circulating factors, revealing a number of previously unappreciated secreted proteins with strong associations to physiological states such as aging^[15–17]^, metabolic status^[18–20]^, and inflammation^[21]^. Particularly, tissue-specific secretome profiling strategies, such as proximity labeling with promiscuous biotin ligases targeted to the endoplasmic reticulum, have further enabled *in vivo* mapping of tissue-specific secreted proteins in model organisms^[22–26]^.

Despite rapid progress in secretome discovery, understanding how secreted proteins exert their biological functions remains a major challenge. For many secreted proteins, the identity of their receptors or interacting partners in target tissues is still elusive. Historically, receptor identification has relied on biochemical purification, affinity-based pull-down, or functional genetic screening approaches. Although these methods have yielded important findings to date, they are often labor-intensive, require large amounts of material, and exhibit limited sensitivity to weak or transient extracellular interactions. As a result, conventional interaction-mapping strategies often fail to capture physiologically relevant but biophysically fragile interactions. This limitation has motivated the development of new genetic, chemical, and computational approaches aimed at systematically identifying receptors and binding partners for secreted factors.

In this review, we summarize and evaluate current strategies to deorphanize secreted proteins, spanning classical biochemical methods, genetic screening approaches, and emerging computational frameworks (Table 1). We discuss the strengths and limitations of each approach, highlight recent methodological innovations, and propose future directions to overcome existing barriers. By integrating experimental and computational advances, deorphanization of the secretome is poised to transition from a case-by-case endeavor to a scalable and systematic discovery paradigm.

## 2. Approaches to Deorphanize Secreted proteins

### 2.1 Biochemical approaches

#### 2.1.1 Affinity pulldown-mass spectrometry (AP-MS)

AP-MS is a powerful and unbiased approach for elucidating protein-protein interactions (PPIs). Recent technical advances have further expanded the utility of AP-MS, such as the development of high-affinity antibodies and nanobodies for efficient target capture^[27–30]^, Clustered Regularly-Interspaced Short Palindromic Repeats (CRISPR)-based epitope-tagging of endogenous proteins^[31–34]^, and increased sensitivity and depth of modern mass spectrometry platforms^[35,36]^. These improvements have collectively enabled broader application of AP-MS across diverse model systems. Although AP-MS has been widely applied to intracellular interactome mapping, it has also been successfully used to identify extracellular PPIs, including ligand-receptor (L-R) interactions. For example, the glycosylphosphatidylinositol (GPI)-anchored protein, reversion-inducing cysteine-rich protein with Kazal motifs (RECK), was discovered as a binding partner of G protein-coupled receptor 124 (GPR124) that stabilizes WNT7 binding in rodent and human through affinity chromatography-MS^[37]^. Beyond vertebrate systems, AP-MS has also revealed extracellular L-R interactions in invertebrates. For example, Grp78 was identified as an antagonist of the adiponectin receptor (AdipoR) of *Drosophila*^[38]^. In addition, Tollo was identified as a binding partner that activates the adhesion G-protein-coupled receptor (GPCR) Cirl in *Drosophila*^[39]^, and lipoprotein Lipid Transfer Particle (LTP) was discovered to interact with the Lipophorin receptor^[40]^. These studies highlight the potential of AP-MS to uncover physiologically relevant extracellular interactions.

However, affinity-based pull-down approaches remain technically challenging, particularly for discovering extracellular PPIs. Successful co-purification of preys with baits depends on multiple factors, including buffer composition, protein abundance, stability of the protein complex, and the intrinsic binding affinity between interacting partners. As a result, optimizing pull-down conditions is often the most critical and rate-limiting step in AP-MS–based interaction discovery. In addition, detection of low-abundance interacting partners remains challenging despite substantial improvements in MS sensitivity. Finally, careful consideration must be given to epitope tag placement, as improper positioning may disrupt native interactions or interfere with protein functions.

#### 2.1.2. Crosslinking-based receptor capture

Because many extracellular L-R interactions are weak, transient, and readily disrupted during detergent solubilization and affinity purification^[41]^, several groups have developed crosslinking-based receptor capture strategies that stabilize L-R complexes directly on living cells. A prototypical example is TRICEPS^[42]^, a trifunctional reagent that enables ligand-guided receptor identification under near-physiological conditions. In TRICEPS, one reactive group couples to the ligand (commonly through primary amines), a second group reacts with oxidized glycans on cell-surface glycoproteins, and a biotin handle enables enrichment and quantitative MS-based identification of captured receptor peptides. This design allows selective recovery of glycosylated cell-surface receptors engaged by the ligand on intact cells.

To expand the chemical scope and improve performance on living cells, HATRIC-based ligand receptor capture (HATRIC-LRC)^[43]^ was developed, which combines a click-chemistry-based workflow with a water-soluble catalyst, enabling capture of L-R interactions at physiological pH. Mechanistically, HATRIC-LRC relies on hydrazone formation between a HATRIC-conjugated ligand and oxidized glycans on proximal cell-surface receptors, which is accelerated by a water-soluble catalyst at physiological pH. Notably, HATRIC-LRC was shown to be effective across diverse ligand types, from small molecules and antibodies to intact viruses, highlighting its flexibility for receptor deorphanization in native membrane contexts.

More recently, rather than focusing on a single secreted protein, interaction-guided crosslinking (IGC) was developed as a next-generation approach to map interactions between surfaceome and secretome at a global scale^[44]^. In IGC, secreted proteins in conditioned media are first conjugated to trifunctional probes that contain a ligand-coupling group, a crosslinking module, and a biotin enrichment handle. After binding of labeled secretome components to receptors on living cells, L-R complexes are selectively crosslinked *in situ* (via UV irradiation or click chemistry), enriched by streptavidin, and identified by LC-MS/MS. Importantly, IGC markedly improved sensitivity, enabling more systematic mapping of secretome–surfaceome interactions.

Complementary to ligand-engineering approaches, recent advances have focused on engineering receptors to directly capture their interacting ligands. One strategy modifies receptors to carry photoactivatable chemical groups positioned near the ligand-binding interface^[45]^. Upon light activation, these groups form covalent bonds with proximal ligands, thereby stabilizing transient L-R interactions. This approach successfully identified neuropeptide L-LEN as an endogenous ligand of GPR50, demonstrating its utility for deorphanizing GPCR.

Despite their utility, crosslinking-based receptor capture methods have notable technical caveats. First, ligand derivatization can potentially reduce binding activity by altering folding, masking receptor-interacting surfaces, or changing binding kinetics^[46]^, and therefore typically requires careful optimization and appropriate controls. Second, TRICEPS/HATRIC-type workflows are inherently biased toward glycosylated surface proteins and depend on efficient, controlled glycan oxidation and crosslinking chemistry on living cells, which can vary across cell types and experimental conditions.

### 2.2 Genetic approaches

#### 2.2.1 In vivo

Few model organisms, most notably *Drosophila melanogaster* and *Caenorhabditis elegans*, are equipped with large-scale RNA interference (RNAi) resources, including transgenic animal library^[47,48]^ and *E.coli* feeding library^[49]^, respectively, enabling tissue-specific knockdown or overexpression. These resources have made *in vivo* genetic screening, a powerful approach to identify L-R interactions in a physiological context. For example, RNAi-based screening in *Drosophila* led to the identification of Grindelwald (Grnd) as a receptor for eiger, *Drosophila* TNF-a^[50]^. Similar genetic strategies in *C. elegans* have also revealed DCAR-1 as a receptor for fungal 4-hydroxyphenyllactic acid^[51]^. Furthermore, in accordance with recent advances in adeno-associated virus-based targeted gene delivery systems^[52–54]^ and CRISPR-based genetic engineering^[55]^, genetic screening has been emerging as a feasible approach in rodent models as well^[56]^. *In vivo* genetic screening remains one of the most physiologically relevant strategies for identifying L-R interactions.

#### 2.2.2 In vitro

In contrast to *in vivo* approaches, *in vitro* genetic screening is typically performed using pooled screening strategies, such as CRISPR-based perturbation libraries or microarray-based platforms. These approaches enable high-throughput screening in a less labor-intensive manner and are particularly amenable to large-scale receptor identification.

Pooled CRISPR-Cas9 knockout (CRISPR-KO) screening has been widely used to identify receptors whose loss confers resistance or sensitivity to ligand treatment. For example, cellular receptors for bacterial Tc toxin^[57]^ and shrimp toxins^[58]^ were identified through positive selection of CRISPR-KO screens, in which cells lacking the relevant receptor survived toxin exposure. Similarly, CRISPR interference (CRISPRi) screening was used to uncover the CD43 and Siglec-7 pair as a key interaction suppressing immune cell activity by leukemia cells^[59]^. These approaches are effective when ligand treatment induces a clear and selectable cellular phenotype.

For cases in which ligand treatment does not produce an obvious phenotype, few studies alternatively have come up with an approach to fish out receptor-overexpressing cells with recombinant ligand proteins. In this approach, CRISPR activation (CRISPRa) was used to generate cell libraries that overexpress transmembrane proteins. CRISPRa employs nuclease-dead Cas9 (dCas9) fused to transcriptional activators, such as VP64-p65-Rta (VPR)^[60]^, SunTag^[61]^, or synergistic activation mediator (SAM)^[62]^, to induce endogenous gene expression. This system provides a practical alternative to open reading frame (ORF)-based overexpression libraries, which are resource- and labor-intensive to generate. The established cell libraries with CRISPRa were incubated with epitope-labeled recombinant ligand proteins, and fluorescence-activated or magnetic-activated cell sorting isolated the ligand-binding cells^[63–65]^.

#### 2.2.3 Caveats and limitations of genetic approaches

Despite their strengths, genetic screening approaches have several important limitations. Most importantly, genetic screening not only identifies direct interacting target proteins, but also uncovers additional proteins that are functionally linked to the observed phenotypes. *In vivo* screening remains labor-intensive, even in organisms with extensive transgenic resources. Moreover, the phenotypic impact of genetic loss-of-function can be attenuated by potential compensatory or redundant L-R interactions. In *in vitro* CRISPR-KO or CRISPRi screening, a major bottleneck is the requirement for a well-defined and quantifiable cellular phenotype upon ligand treatment. In many cases, measurable phenotypes are limited to cell survival or binary intracellular readouts detectable by antibody staining. Recent development of optical pooled screening allowing image-based analysis of intracellular changes, have expanded the range of phenotypes, which can be interrogated in *in vitro* cell screening, including cellular morphology and subcellular protein localization^[66]^. However, these approaches remain costly and are not yet widely accessible. Although CRISPRa enables programmable overexpression of a wide range of genes of interest, its overexpression efficiency varies substantially depending on target genes and cell types^[62,67]^. This limited overexpression efficiency reduces the likelihood or avidity of L-R interactions and consequently constrains the sensitivity of CRISPRa-based receptor discovery.

### 2.3 Computational approaches

#### 2.3.1 AI-based protein structure modeling

Recent advances in AI-based protein structure modeling have substantially transformed approaches to explore PPIs^[68–70]^. AlphaFold-Multimer^[68]^, RoseTTAFold^[70]^, and AlphaFold3^[69]^ provide quantitative estimates of interaction likelihood among multiple proteins, enabling large-scale *in silico* prediction of PPIs. Importantly, the 5-HT2A receptor structure predicted by AlphaFold-Multimer exhibited comparable structural accuracy to that derived from cryo-EM in ligand discovery, which is sufficient to examine L-R interactions^[71]^. Moreover, by benchmarking AlphaFold-Multimer as a screening tool against single-pass membrane proteins, one study systematically prioritized potential receptors for 50 orphan secreted proteins^[72]^. Furthermore, a recent study systematically predicted interactions between plant pathogen-derived secreted small proteins and plant hydrolases through AlphaFold-Multimer, revealing key PPIs in host-pathogen interactions^[73]^.

Beyond predictions for the customized PPIs subsets, large-scale predictions for putative PPIs including L-R interactions have rapidly expanded by leveraging these tools. Indeed, public databases now contain approximately more than 1.6 million human PPI predictions^[74,75]^, as well as 800,000 predicted *Drosophila* PPI pairs^[76]^ generated using AlphaFold-based pipelines. These large datasets of predicted PPIs can be invaluable resources when combined with transcriptional expression profiles across cell types and tissues. Several such resources have been released for diverse model systems such as the Human Protein Atlas^[77,78]^, mouse brain atlas^[79]^, single-cell atlas of *Drosophila melanogaster*^[80]^, and *Caenorhabditis elegans*^[81]^. Similar filtering strategies can also be extended using tissue-specific secretome datasets generated by proximity labeling approaches, such as ER-localized TurboID^[22–25]^ and biotin identification (BioID)^[26]^ or horseradish peroxidase (HRP)-based surface interactome mapping^[82,83]^, further refining candidate interactions based on ligand and receptor availability in their tissue origin.

#### 2.3.2 Caveats and limitations

Despite its speed and scalability, AlphaFold-based deorphanizing L-R pairs has several important limitations. First, many secreted proteins are produced as propeptides and undergo proteolytic cleavage during transit through the secretory pathway^[84]^. Although computational tools exist to predict cleavage sites, including ProP^[85]^, our knowledge on the exact mature forms of many secreted proteins remains incomplete. Without accurate information on the cleaved, biologically active protein sequence, the predictive power of AlphaFold is substantially reduced.

Second, extracellular proteins frequently undergo extensive post-translational modifications, including glycosylation^[86]^, disulfide bond formation^[87]^, and palmitoylation^[88]^, which can critically influence L-R interactions. However, AlphaFold-Multimer^[68]^ and RoseTTAFold^[70]^ accept only amino acid sequences and do not account for post-translational modifications. While AlphaFold3^[69]^ and RoseTTAFold All-Atom^[89]^ allow limited modeling of certain modifications including glycosylation, the diversity and complexity of extracellular glycosylation patterns remain largely intractable with computational testing. This limitation is particularly noteworthy given growing evidence that glycans can actively mediate or stabilize extracellular interactions^[90]^, especially in immune signaling^[91,92]^.

Finally, scoring and confidence assessment of predicted L-R interactions remain underdeveloped. Several metrics, including interface pTM (ipTM)^[68]^, Predicted DockQ score (pDockQ)^[93]^, Predicted DockQ version 2 (pDockQ2)^[94]^, Local Interaction Score (LIS)^[76]^, and interface-aware scoring framework (iLIS)^[95]^, have been proposed to evaluate interaction feasibility, but no consensus cutoff that reliably distinguishes true positives from false positives or negatives exists yet.

### 2.4 Future directions

#### 2.4.1 Multiplexed screening platforms

Although *in vivo* genetic screening enables identification of L-R interactions based on ligand-driven phenotypes, it is highly labor-intensive and time-consuming. Pooled CRISPR *in vitro* screening partially alleviates this limitation by allowing genome-wide interrogation in a single experiment. However, most existing screening strategies are still designed to identify individual L-R pairs, typically testing one ligand at a time. To accelerate systematic deorphanization of the secretome, next-generation multiplexed screening platforms capable of identifying multiple L-R pairs simultaneously are needed. This gap can be addressed by performing a single experiment that combines receptor and ligand libraries, substantially reducing experimental time, and enabling more comprehensive mapping of L-R interactome. To accomplish this approach, it is essential to establish a direct and scalable linkage between ligands and their cognate receptors in a pooled setting, where both interaction partners are initially unknown. Unlike targeted strategies, multiplexed formats require approaches to record or preserve interaction events with sufficient specificity to allow downstream identification. Potential solutions may involve proximity-based labeling or barcoding strategies coupled to high-throughput sequencing. Addressing these challenges will be critical for establishing multiplexed screening platforms for unbiased, network-level discovery of L-R interactions.

#### 2.4.2 Improved sensitivity for ligand detection

Binding affinities of L-R interactions span a wide range, typically from nanomolar to micromolar dissociation constants (Kd)^[41]^, and many extracellular interactions are transient in nature^[41]^. These properties often preclude stable capture of L-R complexes using conventional biochemical approaches such as AP-MS. To overcome this limitation, strategies that increase effective binding avidity or enable covalent capture of interacting partners can be introduced.

One approach is the avidity-based extracellular interaction screen (AVEXIS), which enhances detection sensitivity by multimerizing soluble recombinant proteins to stabilize weak extracellular interactions^[96]^. Using AVEXIS, 20,503 pairwise combinations of *Drosophila* immunoglobulin superfamily (IgSF) and leucine-rich repeat (LRR) family proteins were tested and identified 83 previously unrecognized PPIs even possessing micromolar Kd^[97]^. This study demonstrated that avidity-based strategies can substantially improve detection of low-affinity extracellular interactions.

In addition to avidity enhancement, several bioconjugation technologies have been developed to covalently capture transient interactions. For example, the SpyTag-SpyCatcher system^[98]^ and its engineered variants (SpyTag002-SpyCatcher002 and SpyTag003-SpyCatcher003)^[99,100]^ enable spontaneous and irreversible isopeptide bond formation upon proximity, thereby stabilizing transient PPIs. Similarly, split inteins can reconstitute a full-length protein through protein splicing when two interacting protein fragments are brought into close proximity^[101]^. Sortase A-mediated ligation provides another covalent strategy, in which the enzyme recognizes an Leu-Pro-X-Thr-Gly (LPXTG) motif and catalyzes site-specific transpeptidation between threonine and glycine residues^[102]^. By introducing two bioconjugation components into ligands and receptors, L-R interactions can be stably captured in following experimental steps. Together, these approaches offer powerful means to enhance sensitivity for detecting weak or transient L-R interactions.

#### 2.4.3 Tools for measuring direct ligand-receptor binding

During *in vitro* cell screening, ligand-driven cellular phenotypes must be characterized to identify its cognate receptor. However, these phenotypes vary significantly depending on cell type, and many newly identified secreted proteins have not yet been functionally characterized. Previous CRISPRa screenings have attempted to solve this challenge by treating epitope-labeled recombinant proteins and isolating binding cells through fluorescence-activated cell sorting (FACS) or magnetic bead-based selection (MACS)^[63–65]^. While successful in some cases, these approaches rely heavily on binding affinity. Because many physiological L-R interactions are transient or weak^[41]^, a substantial number of interactions can be lost during the rigorous washing steps of the screening process. Furthermore, ligand binding often induces receptor endocytosis, preventing the stable capture of these interactions on the plasma membrane.

To overcome these limitations, it is necessary to develop tools that convert transient L-R interactions into a stable, amplified genetic signal. Recent breakthroughs in synthetic biology have provided modular toolkits, such as synthetic Notch (synNotch)^[103]^, Programmable Antigen-gated G-protein-coupled Engineered Receptors (PAGER)^[104]^, and Juxtacrine-controlled Universal Programmable Interaction-dependent Transcriptional Effector Reporter (JUPITER)^[105]^, to achieve this transition. By repurposing these systems as “contact-reporting” sensors, a more robust deorphanization pipeline can be achieved. SynNotch and the JUPITER systems are designed to detect cell-to-cell (juxtacrine) interactions. In synNotch, mechanical tension from ligand binding triggers the proteolytic release of a synthetic transcription factor^[103]^. Similarly, JUPITER offers an improved PPI detection system by coupling high-affinity binder and transcriptional juxtacrine sensors in yeast^[105]^. By tethering an orphan ligand or receptor to the extracellular sensing domain of these receptors, a “Receiver” cell could be engineered to remain transcriptionally silent until it physically contacts a “Sender” cell expressing the cognate receptor or membrane-tethered ligand. When integrated with CRISPR libraries, these reporters should allow high-throughput identification of partners, as even a brief binding event can trigger a permanent fluorescent or selectable marker (e.g., green fluorescent protein (GFP) or antibiotic resistance), solving the problem of transient affinity and receptor endocytosis.

While synNotch and JUPITER excel at mapping membrane-bound pairs, the PAGER system addresses the critical challenge of deorphanizing soluble ligands. PAGER utilizes a drug-gated auto-inhibitory mechanism where receptor activation is blocked by a tethered antagonist until the specific ligand binds to the receptor^[104]^. This system is particularly valuable for deorphanizing secreted neuropeptides or cytokines. Because PAGER is based on GPCR architecture and sensitive to soluble factors, it provides a low-background, high-sensitivity readout for ligands that do not require cell-to-cell contact to function.

#### 2.4.4 Integration with spatial and single-cell technologies

Identifying L-R pairs is not just extension of PPIs catalogs but is essential for understanding systemic inter-organ communication. To elucidate the biological function of a L-R interaction, it is necessary to determine where the ligand is produced and secreted, as well as where and in which cell types the cognate receptor is expressed. Without this contextual information, L-R interactions remain difficult to interpret at the physiological level.

Over the past decade, single-cell and single-nucleus transcriptomic technologies^[106]^ have transformed our ability to analyze gene expression across diverse cell types and tissues. By leveraging these approaches, transcriptional profiles can be obtained simultaneously across millions of individual cells, enabling systematic prioritization of target tissues and cell populations relevant to specific L-R interactions. Importantly, these datasets allow investigators to move beyond pairwise interactions and consider how L-R signaling is embedded within broader cellular networks. L-R engagement often initiates downstream transcriptional programs, leading receptor-expressing cells to produce secondary secreted factors or signaling molecules that act on additional tissues or cell types^[107]^. As a result, physiological outcomes frequently arise from cascades of interlinked signaling events rather than from isolated L-R pairs. Capturing this complexity requires integrative frameworks that consider multiple L-R interactions simultaneously.

To address this need, several computational tools have been developed to infer cell-to-cell and inter-organ communication networks from single-cell RNA sequencing data, including CellPhoneDB^[108]^, SingleCellSignalR^[109]^, FlyPhoneDB^[110]^, NicheNet^[111]^, CellChat^[112,113]^, Network Analysis Toolkit for Multicellular Interactions (NATMI)^[114]^ and FlyPhoneDB2^[115]^. These platforms systematically integrate ligand and receptor expression patterns across cell types to predict signaling relationships in a context-dependent manner. Especially, FlyPhoneDB2 combines AlphaFold-predicted extracellular PPIs with single-cell transcriptomic data from *Drosophila* to prioritize L-R pairs in a cell-type-specific manner^[115]^. In parallel, spatial transcriptomic technologies such as multiplexed error-robust fluorescence in situ hybridization (MERFISH)^[116]^, spatially-resolved transcript amplicon readout mapping (STARmap) PLUS^[117]^, and Slide-seq^[118]^ provide spatial resolution on top of transcriptional information, enabling inference of L-R interactions within their native tissue architecture. Recently, a method, LARIS, further extended L-R analysis to spatial transcriptomic datasets^[119]^, facilitating the study of signaling interactions *in situ*.

Together, integration of L-R discovery with single-cell and spatial transcriptomic technologies enables a shift from identifying individual interaction pairs to reconstructing coordinated signaling networks that underlie inter-organ communication. In this context, deorphanizing even a single L-R pair can have broad impact by anchoring larger signaling circuits within defined cellular and spatial frameworks.

## 3. Conclusion

Deorphanizing secreted proteins is essential for understanding systemic signaling and inter-organ communication but remains a central challenge due to the nature of extracellular interactions and the contextual complexity of L-R interplay. In this review, we highlight how recent methodological advances have expanded the toolkits for receptor discovery while also underscoring persistent limitations in sensitivity, scalability, and physiological interpretation. Emerging strategies that integrate biochemical stabilization, screening scalability, computational prioritization, and spatially resolved transcriptomic context offer a path toward more comprehensive and biologically meaningful L-R mapping. Development of hybrid and multiplexed platforms will be critical to move beyond one-to-one discoveries toward network-level reconstruction of secreted signaling pathways.

## Figures and Tables

**Table 1.** Overview of approaches deorphanizing secreted proteins.

	Tools	Advantages	Limitations
**Biochemical approaches**			
AP-MS*		Unbiased identification of interacting partners; directly identifies physical complexes	Weak or transient extracellular interactions often lost; sensitive to buffer conditions and tag placement
Crosslinking-based	TRICEPS,	Capture transient ligand-receptor interaction; superior sensitivity for low-abundance interactions; captures interactions *in situ*	Requires ligand derivatization; biased toward glycosylated surface proteins
receptor capture	HATRIC-LRC^†^		
	IGC^‡^		
**Genetic approaches**			
*In vivo* screening	RNAi screening	Conducted in physiological context	Labor-intensive; limited throughput; particularly powerful only in *Drosophila melanogaster* and *C. elegans*
*In vitro* screening	Microarray	High throughput, genome-wide, unbiased	Requires purified proteins; lacking native membrane context; restricted screenable cellular phenotypes
	CRISPR-knock out		
	CRISPR interference		
	CRISPR activation		
**Computational Approaches**	AlphaFold-Multimer	Rapid, scalable protein-protein interaction prediction; quantitative interaction confidence metrics	Does not account for PTMs or proteolytic processing; uncertain confidence thresholds
	AlphaFold3		
	RosettaFold		
	RoseTTAFold All-Atom		

AP-MS*: Affinity pulldown-mass spectrometry; HATRIC-LRC†: HATRIC-based ligand receptor capture; IGC‡: interaction-guided crosslinking; RNAi: RNA interference; CRISPR: Clustered Regularly-Interspaced Short Palindromic Repeats.
